# Anesthetic Management of a Patient with a Giant Pericardial Cyst Compressing the Right Atrium

**DOI:** 10.1155/2019/2320879

**Published:** 2019-05-26

**Authors:** Mohammad Hadi Gharedaghi, Saman Ahmadi, Arjang Khorasani, Farzad Ebrahimi

**Affiliations:** ^1^Department of Anesthesiology, Advocate Illinois Masonic Medical Center, 836 W Wellington Ave, Suite 4815, Chicago, IL 60657, USA; ^2^Department of Anesthesiology, University of Illinois at Chicago, Chicago, IL, USA

## Abstract

Pericardial cysts are rare mediastinal cysts composed of a single fluid-filled mesothelial layer and can be congenital in origin or develop secondary to pericarditis, trauma, or infection. Although most pericardial cysts are asymptomatic, life-threatening complications can occasionally occur. We report on a 57-year-old man with an asymptomatic 9 cm pericardial cyst that was incidentally found as an abnormal cardiac silhouette on routine chest radiography. Further imaging confirmed the presence of a pericardial cyst that was compressing the right atrium. The patient underwent successful video-assisted thoracoscopic removal of the pericardial cyst under general anesthesia. The patient's postoperative course was uneventful and he was discharged on postoperative day 1 in a stable condition. To our knowledge, this is the first report regarding the anesthetic management of a patient with a giant pericardial cyst undergoing thoracic surgery. Knowledge regarding the perioperative challenges associated with the removal of pericardial cysts can prevent complications and improve patient outcomes.

## 1. Introduction

Pericardial cysts are rare entities and comprise 7% of all mediastinal masses. The reported incidence of pericardial cysts is 1 in 100,000 population per year [1, 2]. These cysts are usually detected in individuals in their third or fourth decade of life and there is no difference in the incidence of pericardial cysts between males and females [3]. Pericardial cysts are commonly congenital. However, pericarditis, bacterial or parasitic infection, trauma, and cardiac surgery may also result in formation of pericardial cysts [4].

The treatment options for pericardial cysts include observation, percutaneous drainage, and surgical resection. Surgical excision is indicated for symptomatic cysts or large asymptomatic cysts given that they are associated with an increased risk of complications [5]. Potential complications of pericardial cysts include compression of the heart, syncope, cardiac tamponade, bronchial compression, and sudden death [6–9]. Video-assisted thoracoscopic surgery (VATS) is a valuable intervention for resection of pericardial cysts with lower morbidity and mortality rates than thoracotomy [5]. The duration of surgery and postoperative hospital stay after intrathoracic cyst resection is lower in patients who undergo VATS in comparison to those who undergo thoracotomy. Moreover, intra- and postoperative complication rates and intensive care unit admission rates are similar between VATS and thoracotomy. Notwithstanding the forgoing, intact resection of large intrathoracic cysts may require performing a thoracotomy [10].

There are several case reports on patients with pericardial cysts in the literature [1, 5–9]. However, the unanticipated challenges that anesthesiologists might face when caring for these patients in the operating room have not been addressed. Herein, we present a patient with a giant pericardial cyst compressing the right atrium who underwent VATS excision. We also discuss the perioperative anesthetic management of this patient.

## 2. Case Presentation

**Figure 1:**
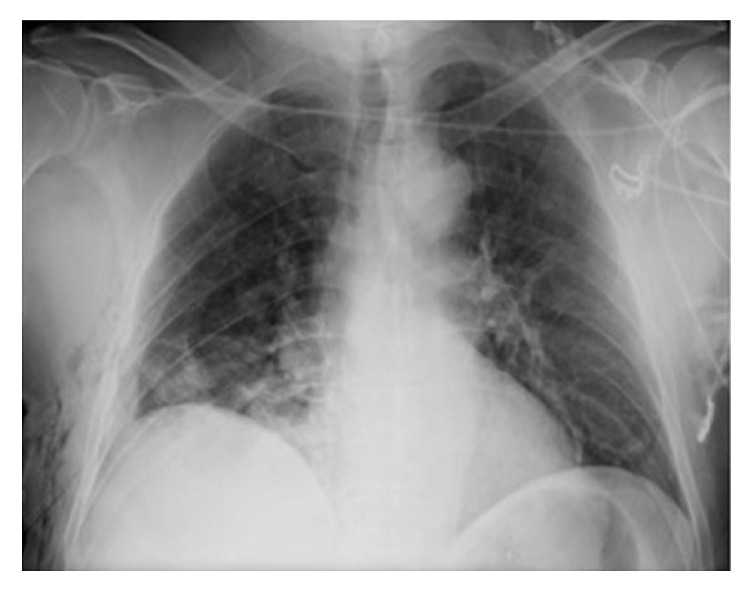
Chest radiography shows an enlarged cardiac silhouette.

**Figure 2:**
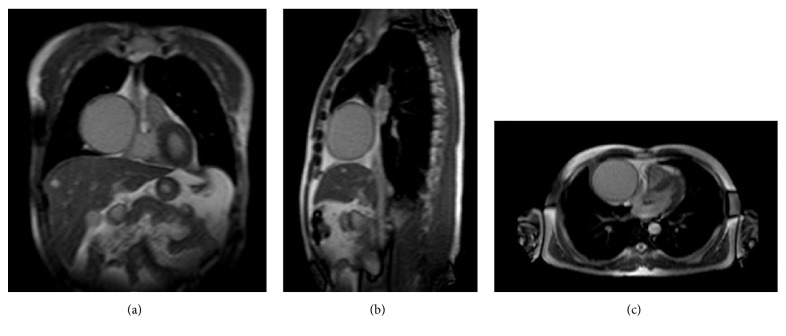
Coronal (a), sagittal (b), and transverse (c) views of thoracic MRI show a 9 cm pericardial cyst located at the right cardiophrenic angle compressing the right atrium.

A 57-year-old asymptomatic man with no significant past medical history was found to have an enlarged cardiac silhouette on a routine chest radiograph (Figure 1). Magnetic resonance imaging (MRI) revealed a 9 cm pericardial cyst in the right cardiophrenic angle that was associated with right atrial compression (Figures 2(a), 2(b), and 2(c)). Although the pericardial cyst wall showed contrast uptake, no uptake within the cyst was observed on first-pass or delayed images. There was no compression of the airway or superior vena cava (SVC) and the pericardial cyst had not eroded into the heart. The patient was not at high risk for hydatid cysts and he did not have any history of fever, suggesting that an infectious cause for his pericardial cyst is unlikely. He did not have any history of chest trauma or intrathoracic surgery. The absence of hypertension, hematuria, and a positive family history made a diagnosis of autosomal dominant polycystic kidney disease (ADPKD) unlikely. The patient was scheduled for resection of the pericardial cyst using VATS. Preoperative electrocardiographic findings, complete blood count results, serum creatinine levels, liver function tests, and serum electrolyte levels were normal.

**Figure 3:**
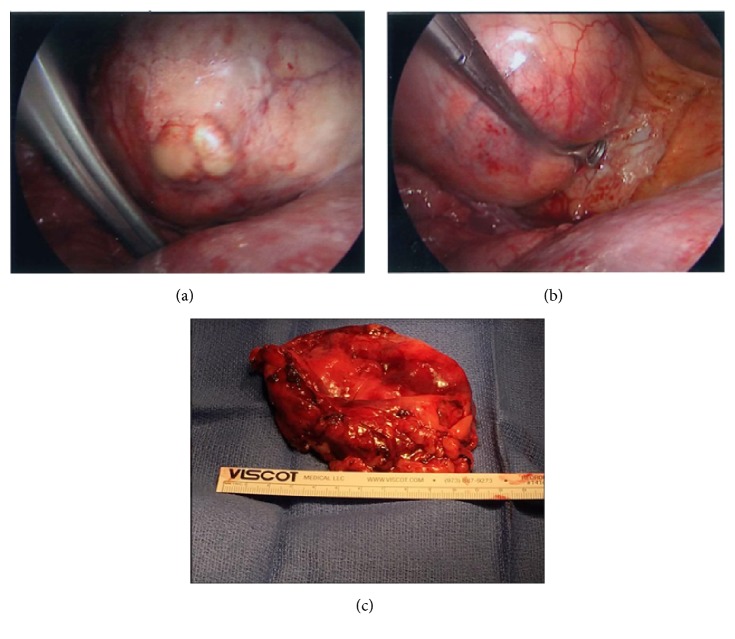
Intraoperative images show the pericardial cyst before (a and b) and after (c) resection.

**Figure 4:**
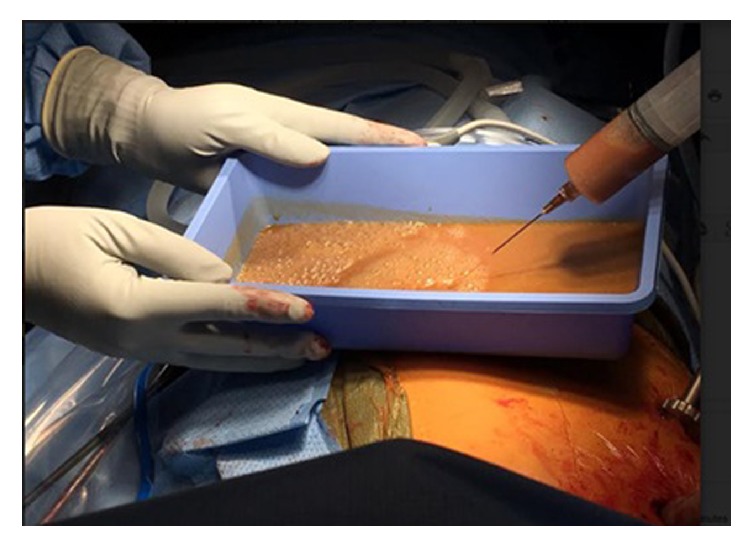
Brown murky fluid aspirated from the pericardial cyst.

On the day of surgery, the physical exam, including heart and lung auscultation, was unremarkable and the vital signs were within normal limits (blood pressure of 119/75 mmHg, heart rate of 83 beats per minute, respiratory rate of 14 per minute, blood oxygen saturation of 97% on room air, and temperature of 36.9°C). A left radial arterial line and two large-bore intravenous catheters were placed. The patient was adequately hydrated with intravenous administration of normal saline. He was transferred to the operating room and placed in the supine position on the operating table. The standard American Society of Anesthesiologists monitors were placed on the patient. The pericardial cyst did not compress the patient's right bronchus or the SVC, and therefore, he was able to tolerate the supine position with no shortness of breath or hemodynamic instability. The patient was preoxygenated and general anesthesia was induced by slow intravenous administration of etomidate 0.2 mg/kg and fentanyl 1 *μ*g/kg. Neuromuscular blockade was achieved by intravenous administration of succinylcholine 1.5 mg/kg. Ephedrine 0.1 mg/kg was administered following induction to minimize the hemodynamic effects of the induction agents and positive pressure ventilation. A 37-French left-sided double-lumen endobronchial tube (DLT) was placed. The patient was placed in the left lateral decubitus position. Anesthesia was maintained with oxygen (fraction of inspired oxygen of 0.6), air, and sevoflurane (1 minimum alveolar concentration). The right lung was collapsed and neuromuscular blockade was induced with rocuronium 0.6 mg/kg. A 1-cm incision was made in the posterior axillary line at the fifth intercostal space. A metal port was placed and a 10-mm, 30° thoracoscope was placed through the incision. A 2-cm incision was made above the fifth intercostal space at the mid axillary line to access the cyst. The pericardial cyst was found to be firm, and had some calcifications on the surface (Figures 3(a), 3(b), and 3(c)). The cyst was easily separated from the pericardial fat. However, it was attached to the anterior chest wall. Following separation of the cyst from the anterior chest wall some bleeding occurred from the distal right internal mammary artery. The bleeding was controlled with Enseal and clips. The cyst was large, and hence, access to the superior aspect of the cyst was difficult. Therefore, an 18-gauge needle was used to aspirate the cyst fluid. Approximately 300 ml of brown, murky, nonodorous fluid was aspirated from the cyst before it was completely resected (Figure 4). The patient remained hemodynamically stable throughout the procedure and the DLT was removed in the operating room at the end of the procedure. Postoperative pain was managed with an intercostal nerve block using 10 ml of 0.5% bupivacaine and patient controlled analgesia pump using hydromorphone (intravenously, 0.2 mg every 10 minutes). The patient's postoperative course was uneventful, and he was discharged on postoperative day 1 in a stable condition. Cyst fluid cultures were negative.

## 3. Discussion

MRI is considered to be the best modality for the diagnosis and follow-up of a pericardial cyst because it provides excellent delineation of the pericardial anatomy and can aid in the precise localization and characterization of various pericardial lesions. It has higher accuracy than computed tomography for distinguishing malignant tissues from nonmalignant fluid-filled cysts [11]. Pericardial cysts are more commonly located in the right cardiophrenic angle. Therefore, compression of anatomical structures located on the right side of the heart, such as the SVC, right atrium, right ventricle, right main bronchus is more commonly seen as a result of pericardial cysts. In the rare event of a left pericardial cyst, compression of the left atrium and ventricle might occur [12]. The size of pericardial cysts usually varies from 2 to 5 cm [7]. In our case, MRI revealed a large 9-cm pericardial cyst in the right cardiophrenic angle that was compressing the right atrium (Figures 2(a), 2(b), and 2(c)).

It is important for anesthesiologists to understand the physiological alterations caused by the presence of a giant pericardial cyst, given that they may have to anesthetize patients with this rare cyst.

### 3.1. Preoperative Considerations in Patients with a Pericardial Cyst

Some reports have demonstrated an association between a pericardial cyst and ADPKD. Given the progressive impairment of renal function in patients suffering from ADPKD, it is imperative to assess kidney function in patients with pericardial cysts who are suspected of having ADPKD before surgery [13]. Although pericardial hydatid cysts are rare, elevated levels of immunoglobulin E, the presence of eosinophilia and increased serum levels of liver enzymes suggest the diagnosis of hydatid cyst in high-risk patients with intrathoracic cysts [14].

A pericardial cyst may cause significant compression of the SVC and right atrium and lead to a reduction of preload and cardiac output [15]. This necessitates significant fluid resuscitation before the induction of anesthesia. Large-bore intravenous lines in the lower extremities are recommended in patients with compression of the SVC [16]. Lung isolation during surgery, proximity of the cyst to major vessels and the risk of right ventricular outflow tract erosion in rare cases necessitate preoperative placement of an arterial line. Finally, the possibility of airway collapse means that heavy sedation should be avoided and premedication should be limited to antisialagogues [4, 17].

### 3.2. Intraoperative Considerations in Patients with a Pericardial Cyst

Patient positioning can be challenging. Semirecumbent or sitting positions can prevent an increase in intracranial pressure in a patient with compression of the SVC. Orthopnea or a sudden decrease in oxygen saturation following patient positioning is suggestive of airway obstruction [18]. Whenever airway compression exists secondary to a pericardial cyst, the right main bronchus is the most common location of obstruction [4]. Induction of general anesthesia has been found to exacerbate extrinsic airway compression by decreasing the negative intrathoracic pressure and relaxing the bronchial smooth muscles. Airway compression can be further exacerbated by neuromuscular blockade and positive pressure ventilation, which reduce the normal trans-pleural gradients and cause narrowing of the large-caliber airways [19]. Awake fiberoptic endotracheal intubation or inhalational induction in the sitting position maintain spontaneous ventilation, prevent positional airway compression by the pericardial cyst and preserve negative intrathoracic pressures. For this reason the aforementioned induction techniques seem to be plausible options for these patients [16]. Use of a single-lumen endotracheal tube with a bronchial blocker is a suitable alternative to a DLT for patients undergoing awake fiberoptic endotracheal intubation. In our patient, no airway compression was observed on MRI. Neuromuscular blockade was induced by succinylcholine to shorten the effects of positive pressure ventilation on the patient's hemodynamics in response to the compressed right atrium [16]. However, when one lung was isolated by the DLT and the right lung collapsed, rocuronium was administered and positive pressure ventilation was restarted. From a theoretical standpoint, collapsing the right lung creates more free space in the intrathoracic cavity and relieves any right atrial compression caused by the pericardial cyst. Therefore, we did not expect significant hemodynamic changes after the initiation of one-lung ventilation.

Hypotension may occur during induction in response to decreased systemic vascular resistance with a fixed cardiac output or venodilatation. Hypotension is further exacerbated if the right atrium or right ventricles are compressed by the pericardial cyst. Hence, drugs that cause venodilatation or a fall in systemic vascular resistance should be used with caution. Ketamine can be used safely as an induction agent due to its sympathomimetic properties [20]. We used etomidate as an induction agent in our patient because of its minimal hemodynamic effects. We also used ephedrine during induction to increase vasoconstriction and cardiac output. A pericardial cyst is mobile within the thorax, so certain positions may cause obstruction of the SVC or ventricular outflow tract [21]. To a certain extent, this can be assessed preoperatively because patients are more symptomatic in those positions. Nevertheless, careful monitoring of the hemodynamics during positioning and a titrated induction technique should always be employed. In addition, all arrangements for initiation of emergent cardiopulmonary bypass should be made before induction of anesthesia to avoid catastrophic situations [22].

Supraventricular arrhythmias, such as atrial fibrillation, are not uncommon in patients with a pericardial cyst [23]. Interestingly, palpitation due to cardiac arrhythmia can be the first presenting symptom of this disease. Compression of the atria, pulmonary veins or the sinoatrial node region can be the predisposing factors for the cardiac dysrhythmia [23]. It is important to address any correctable precipitating factor such as hypovolemia, hypoxia, or electrolyte imbalance perioperatively.

Erosion of the right ventricular wall and SVC by a pericardial cyst has been reported [4, 15, 17, 24]. This significantly increases the risk of bleeding during excision of the cyst. Although removal of pericardial cysts that only cause compression and not erosion can be achieved without cardiopulmonary bypass, it is highly recommended that the cardiopulmonary bypass machine be available on standby when erosion into the cardiac structures is present. Placement of a central venous line can be used to monitor the central venous pressure (CVP). If the pericardial cyst compresses the right ventricular outflow tract, a reduction of CVP can be appreciated following cyst removal [25]. In addition, a central venous line can be used for the purpose of resuscitation and vasopressor administration.

Excision of large pericardial cysts can be very challenging. In our case, the cyst fluid was aspirated to help the surgeon with the excision (Figure 4). However, perforation of the cyst and intrathoracic contamination by the fluid can be dangerous and life-threatening when the cyst is infected. Severe allergic reactions and anaphylactic shock have been reported following rupture of a hydatid cyst [26]. Furthermore, vascularized connective tissue in the cyst wall and its frailty increases the risk of massive hemorrhage into the cyst or pericardium. This may lead to cardiac tamponade during surgical removal of the pericardial cyst [27].

### 3.3. Postoperative Considerations in Patients with a Pericardial Cyst

The morbidity and mortality risks following pericardial cyst excision are very low. There is no report of complications during tracheal extubation. All reported cases were extubated following surgery with no complications in the postanesthesia care unit. In the case of video-assisted thoracoscopic cyst excision, pain control can be achieved using acetaminophen, nonsteroidal anti-inflammatory drugs or intravenous opioids. Thoracic epidural analgesia may be required when thoracotomies are performed. Thoracic paravertebral nerve block and serratus anterior plane block can also be used for the purpose of pain control [28, 29].

Atrial fibrillation following thoracic surgery is not uncommon. It is important to rectify all triggering factors [23]. Hypoxia secondary to atelectasis can occur following thoracic surgery and needs to be ruled out as a triggering factor for postoperative cardiac arrhythmias.

It is very important that anesthesiologists be aware of the perioperative challenges associated with pericardial cysts. This knowledge can prevent complications and improve patient outcomes.

## References

[1] Seo G. W., Seol S., Jeong H. (2014). A large pericardial cyst compressing the left atrium presenting as a pericardiopleural efussion. *Heart, Lung and Circulation*.

[2] Hynes J. K., Tajik A. J., Osborn M. J., Orszulak T. A., Seward J. B. (1983). Two-dimensional echocardiographic diagnosis of pericardial cyst. *Mayo Clinic Proceedings*.

[3] Siti Salwa M. S., Anas R., Nor Hidayah A. B. (2013). Pericardial cyst: A rare cause of pericardial effusion. *Medical Journal of Malaysia*.

[4] Kar S. K., Ganguly T., Dasgupta S., Mitra M., Bhattacharya R. (2015). Pericardial cyst: a review of historical perspective and current concept of diagnosis and management. *Interventional Cardiology Journal*.

[5] Makar M., Makar G., Yousef K. (2018). Large pericardial cyst presenting as acute cough: a rare case report. *Case Reports in Cardiology*.

[6] Bandeira F., Desa V., Moriguti J. (1996). Cardiac tamponade: An unusual complication of pericardial cyst. *Journal of the American Society of Echocardiography*.

[7] Martins I. M., Fernandes J. M., Gelape C. L., Braulio R., Silva Vde C., Nunes Mdo C. (2011). A large pericardial cyst presenting with compression of the right-side cardiac chambers. *Revista Brasileira de Cirurgia Cardiovascular*.

[8] Davis W. C., German J. D., Johnson N. J. (1961). Pericardial diverticulum causing pulmonary obstruction. *Archives of Surgery*.

[9] Fredman C. S., Parsons S. R., Aquino T. I., Hamilton W. P. (1994). Sudden death after a stress test in a patient with a large pericardial cyst. *American Heart Journal*.

[10] Ulaş A. B., Aydin Y., Eroglu A. (2018). Comparison of video-assisted thoracoscopic surgery and thoracotomy in the treatment of mediastinal cysts. *Turkish Journal of Thoracic and Cardiovascular Surgery*.

[11] Raja A., Walker J. R., Sud M. (2011). Diagnosis of pericardial cysts using diffusion weighted magnetic resonance imaging: A case series. *Journal of Medical Case Reports*.

[12] Feigin D. S., Fenoglio J. J., McAllister H. A., Madewell J. E. (1977). Pericardial cysts. A radiologic-pathologic correlation and review. *Radiology*.

[13] Hooda A. K., Narula A. S. (2005). Pericardial cyst: a rare association of autosomal dominant polycystic kidney disease. *Indian Journal of Nephrology*.

[14] Kumar Paswan A., Prakash S., Dubey R. K. (2014). Cardiac tamponade by hydatid pericardial cyst: a rare case report. *Anesthesiology and Pain Medicine*.

[15] Kaul P., Javangula K., Farook S. A. (2008). Massive benign pericardial cyst presenting with simultaneous superior vena cava and middle lobe syndromes. *Journal of Cardiothoracic Surgery*.

[16] Ku C. M. (2011). Anesthesia for patients with mediastinal masses. *Principles and Practice of Anesthesia for Thoracic Surgery*.

[17] Chopra P. S., Duke D. J., Pellett J. R., Rahko P. S. (1991). Pericardial cyst with partial erosion of the right ventricular wall. *The Annals of Thoracic Surgery*.

[18] O'Leary H. T., Tracey J. A. (1983). Mediastinal tumours causing airway obstruction. A case in an adult.. *Anaesthesia*.

[19] Sibert K. S., Biondi J. W., Hirsch N. P. (1987). Spontaneous respiration during thoracotomy in a patient with a mediastinal mass. *Anesthesia & Analgesia*.

[20] Frawley G., Low J., Brown T. C. K. (1995). Anaesthesia for an anterior mediastinal mass with ketamine and midazolam infusion. *Anaesthesia and Intensive Care*.

[21] Russell J. C., Lowry K. G. (2003). Presentation of non-Hodgkin's lymphoma as acute hypoxia caused by right ventricular compression. *Anesthesia & Analgesia*.

[22] Tempe D. K., Arya R., Dubey S. (2001). Mediastinal mass resection: Femorofemoral cardiopulmonary bypass before induction of anesthesia in the management of airway obstruction. *Journal of Cardiothoracic and Vascular Anesthesia*.

[23] Generali T., Garatti A., Gagliardotto P., Frigiola A. (2011). Right mesothelial pericardial cyst determining intractable atrial arrhythmias. *Interactive CardioVascular and Thoracic Surgery*.

[24] Mastroroberto P., Chello M., Bevacqua E., Marchese A. R. (1996). Pericardial cyst with partial erosion of the superior vena cava. An unusual case. *The Journal of Cardiovascular Surgery*.

[25] Ng A. F., Olak J. (1997). Pericardial cyst causing right ventricular outflow tract obstruction. *The Annals of Thoracic Surgery*.

[26] Cimpoesu D., Stoica L., Paulet A., Petris A. (2012). A case of anaphylactic shock due to pericardial hydatid cyst. *Chest*.

[27] Shiraishi I., Yamagishi M., Kawakita A., Yamamoto Y., Hamaoka K. (2000). Acute cardiac tamponade caused by massive hemorrhage from pericardial cyst.. *Circulation*.

[28] Mulder D. S. (1993). Pain management principles and anesthesia techniques for thoracoscopy. *The Annals of Thoracic Surgery*.

[29] Alzahrani T. (2017). Pain relief following thoracic surgical procedures: A literature review of the uncommon techniques. *Saudi Journal of Anaesthesia*.

